# mTOR signaling contributes to motor skill learning in mice

**DOI:** 10.3389/fnmol.2014.00026

**Published:** 2014-04-03

**Authors:** Yan Bergeron, Laure Chagniel, Geneviève Bureau, Guy Massicotte, Michel Cyr

**Affiliations:** Groupe de Recherche en Neurosciences, Département de Biologie Médicale, Université du Québec à Trois-RivièresTrois-Rivières, QC, Canada

**Keywords:** 4EBP1, motor learning, mTOR, P70S6K, rapamycin, rotarod, siRNA

## Abstract

The mammalian target of rapamycin (mTOR) kinase is a critical regulator of mRNA translation and is suspected to be involved in various long-lasting forms of synaptic and behavioral plasticity. However, its role in motor learning and control has never been examined. This study investigated, in mice, the implication of mTOR in the learning processes associated with the accelerating rotarod task. We first observed that the rotarod learning did not alter the levels of total mTOR in the striatum, hippocampus, cerebellum, and anterior cortex of trained mice. However, it increased the levels of phosphorylated mTOR in the striatum and hippocampus exclusively during the first session of training; no change was observed at the second and third sessions. In order to further investigate the potential role of mTOR during motor skill learning, we performed systemic and intrastriatal inhibitions of mTOR using the pharmacological inhibitor rapamycin, as well as a genetic knockdown of striatal mTOR using intrastriatal infusion of mTOR siRNA. These three independent approaches were all associated with a significant reduction in rotarod performances that were reminiscent of impaired consolidation processes. Notably, these treatments did not affect the capacity of mice to execute the pole test, suggesting that mTOR activity was mainly controlling motor learning rather than motor abilities. Moreover, all treatments decreased the levels of phosphorylated 4EBP1 and P70S6K, two molecular downstream targets of mTORC1. Our findings demonstrate that striatal mTOR kinase, via the phosphorylation of 4EBP1 and P70S6K, plays an important role in the cellular and molecular processes involved in motor skill learning.

## INTRODUCTION

When faced to new learning experiences, one fascinating characteristic of the brain is its capacity to modulate neural activities and induced long-lasting modifications in neural circuits. These modifications subsequently influence thoughts, feelings and behaviors (Citri and Malenka, 2008). In the case of motor control and learning, it is known that brain areas such as the basal ganglia, motor cortex, and cerebellum cooperate to enable movement (Lynch, 2004). Recent investigations have shown regional-specific changes in neural activity and morphology during the acquisition of motor tasks in the striatum, a major nucleus input of the basal ganglia (Packard and Knowlton, 2002; Kreitzer and Malenka, 2008). For example, it is believed that the dorsal area of the striatum plays a significant role in the learning processes of motor skilled tasks (Costa et al., 2004; Luft et al., 2004). However, the molecular mechanism responsible for the long-lasting modifications in the dorsal striatum during motor learning is still under extensive investigations.

Interestingly, the mammalian target of rapamycin (mTOR) activity has been noticed to be essential in neuronal development, translation of several mRNAs and synaptic plasticity (Bekinschtein et al., 2007; Swiech et al., 2008; Belelovsky et al., 2009; Richter and Klann, 2009). This serine/threonine kinase forms two complexes, mTORC1 and mTORC2. The first complex plays a major role in regulating critical cellular processes such as cell growth, proliferation, transcription, protein synthesis as well as ribosomal biogenesis by phosphorylating P70S6K and eukaryotic initiation factor 4E (eIF4E)-binding proteins (4EBPs; Sarbassov et al., 2005; Wang and Proud, 2006; Hoeffer and Klann, 2009; Urbanska et al., 2012). The second complex is involved in cytoskeletal organization (Jacinto et al., 2004; Sarbassov et al., 2006). Recently, it has been demonstrated that molecular pathways involving mTOR activity are essential for the long-term potentiation (LTP) and long-term depression (LTD; Gkogkas et al., 2010; Garelick and Kennedy, 2011). Furthermore, behavioral experiments investigating spatial learning, object recognition and inhibitory avoidance memory have recognized a role for mTOR activity (Qi et al., 2010; Jobim et al., 2011a,b). Whether mTOR activity is influenced or engaged during the execution of motor movement and motor learning is totally unknown.

The present study establishes, by using two different pharmacological and one genetic approaches, that mTOR activity of the dorsal striatum is one important molecular step involved in learning consolidation, but not motor abilities, during the acquisition of a complex motor skill in mice. Notably, we demonstrate the implication of two downstream targets of mTORC1 activation, P70S6K and 4EBP1, in this process.

## MATERIALS AND METHODS

### ANIMALS

C57bl/6j male mice of 60–75 days old (Charles River, St-Constant, QC, Canada) were used. They were individually housed in a controlled room (light cycle: 14 h; dark cycle: 10 h) with *ad libitum* access to water and food. All experiments and procedures were approved by the UQTR Institutional Animal Care and Use Committee and performed in accordance with the Canadian Council on Animal Care.

### ROTAROD

To assess motor skill learning, we used the accelerating rotarod. Mice performances on the rotarod (AccuScan Instruments, Columbus, OH, USA), which accelerates from 4 to 40 rpm in 300 s, were evaluated for 10 trials per session on three consecutive days. A resting time of 180 s was allowed between each trial. The end of a trial was considered when mice were falling off the rod or when they reached 300 s. Latency to fall was recorded for each trial.

### POLE TEST

The pole test was performed before the first trial of the third rotarod training day, 15 min after the systemic or intrastriatal rapamycin injections or at the 14th day of siRNA infusion. Briefly, the pole test consist of a vertical rod (Diameter: 1.5 cm; Length: 50 cm) fixed on a table with a tennis ball at its upper end. Mice were placed on the upper end of the rod facing the tennis ball. The time required for each mouse to turn around and descend the vertical rod was recorded. A maximum time of 120 s was allowed to execute the task. To make sure they fully learned the motor task before the recorded test, mice were pre-trained three consecutive times.

### PHARMACOLOGICAL TREATMENTS

**FIGURE 1:**
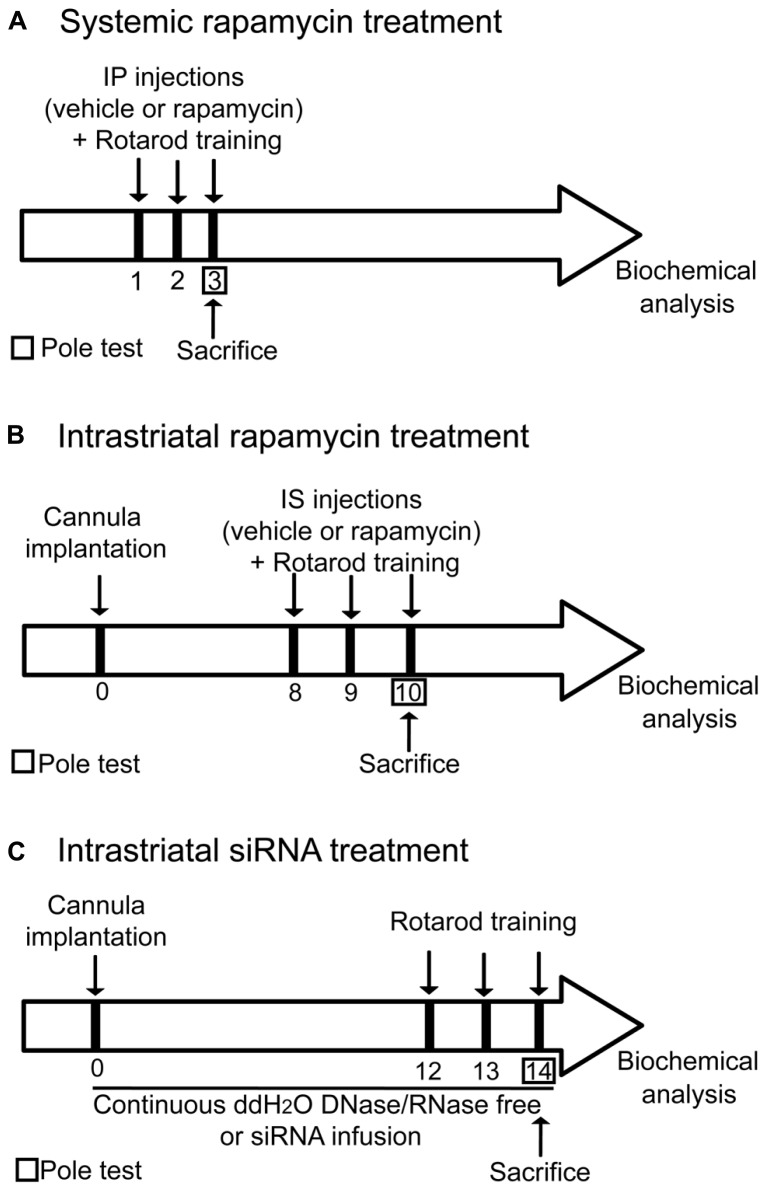
**Illustration depicting the experimental design.** All animals were subjected to the same handling treatment before the experiments. **(A)** For systemic treatment, mice were injected systemically with vehicle (DMSO) or rapamycin 15 min prior each rotarod training session. **(B)** For intrastriatal treatment, a bilateral guide cannula was implanted in the dorsal striatum of mice. After the surgery, seven days of recovery were given to the mice before starting the experiments. 15 min prior the first trial of each rotarod training days, injection of vehicle (DMSO) or rapamycin was delivered into the dorsal striatum of both hemispheres. **(C)** For intrastriatal siRNA infusion, a bilateral connector cannula was installed into the dorsal striatum. Two osmotic mini-pumps were surgically placed subcutaneously on each side of the scapulae and connected to the cannula via a plastic connector. Mice were receiving for 14 consecutive days a continuous intrastriatal infusion with either distillated/deionized water DNase/RNase free or mTOR siRNA. Mice were trained on the rotarod on the 12th, 13th, and 14th days of treatment.

For systemic treatment, mice were weighed and systemically injected with vehicle (100% DMSO, 180–210 μl) or rapamycin (40 mg/kg, dissolved in 100% DMSO, 180–210 μl; LC laboratories, Woburn, MA, USA) 15 min prior each rotarod training days (**Figure 1A**). In order to discriminate motor endurance activity from motor skill learning, an independent cohort of mice were trained on the accelerating rotarod for four consecutive days. At the fourth day, mice received systemic injections of rapamycin or the vehicle before the beginning of the first trial. For intrastriatal treatment, mice were anesthetized under isoflurane, placed in a stereotaxic apparatus and implanted with a bilateral 26-gage guide cannula (Plastics One, Roanoke, VA, USA). The coordinates used for the cannula implantation were: AP: + 0.86 mm; ML ± 1.50 mm; DV: -3.25 mm relative to Bregma (Atlas of Paxinos and Franklin, 2001). After the surgery, 7 days of recovery were given to the mice before starting the experiments. A dummy cannula (Plastics One, Roanoke, VA, USA) was left in place throughout the experiment. Under anesthesia, 15 min prior the first trial of each rotarod training days, the dummy cannula was replaced by the injector. Injection of vehicle (100% DMSO, 1 μl/side) or rapamycin (1 ng/1 μl/side, dissolved in 100% DMSO; LC laboratories, Woburn, MA, USA) was delivered into the dorsal striatum of both hemispheres at a constant flow of 0.5 μl/min by the micro injector pump (Harvard Apparatus, Montréal, QC, Canada; **Figure 1B**).

### siRNA KNOCKDOWN

Mice were anesthetized under isoflurane and positioned in a stereotaxic frame. A bilateral connector cannula (Plastics One) was installed into the dorsal striatum at the following coordinates: AP: + 0.86 mm; ML: ± 1.50 mm; DV: -3.25 mm relative to Bregma (Atlas of Paxinos and Franklin, 2001). It was kept in place with Loctite instant adhesive. Two osmotic mini-pumps (Alzet, Cupertino, CA, USA, Model 1002) were surgically placed subcutaneously on each side of the scapulae and connected to the cannula via a plastic connector. Osmotic pumps were prefilled with either distillated/deionized water DNase/RNase free (EMD Millipore, 6 μl/day) or mTOR siRNA (1.44 nmol/day, diluted in 100% ddH_2_O DNase/RNase free, Sigma-Aldrich, Oakville, ON, Canada) and mice were receiving a continuous intrastriatal infusion for 14 consecutive days. Mice were trained on the rotarod on the 12th, 13th, and 14th days of treatment (**Figure 1C**).

### WESTERN BLOT ANALYSIS

In all cases, mice were sacrificed 15 min after the last trial of their last rotarod training day and brains were immediately removed. The striatum, hippocampus, cerebellum, and anterior cortex were dissected, frozen on dried ice and preserved at -80°C. Protein extractions of each structure were made by homogenizing the tissue in a solution of RIPA buffer containing 65 mM Tris base, 0.15 M NaCl, 0.1% Triton-100x, 0.25% SDS, 1 mM EDTA and protease/phosphatase inhibitors cocktail (Roche, Indianapolis, IN, USA). The Bradford assay (Bio-Rad, Hercules, CA, USA) was used to evaluate protein concentrations. Samples of loading buffer, water, and 40 μg of proteins were boiled at 95°C for 5 min, then loaded on 8, 10, or 15% SDS-PAGE and transferred onto nitrocellulose membranes. Afterward, membranes were blocked in 5% BSA/TBS-Tween 0.1% for 1 h at room temperature and incubated overnight at 4°C with the primary antibodies diluted in 1% BSA/TBS-Tween 0.1%. Antibodies were raised against phosphorylated mTOR at serine 2448 (Cell Signaling Technology, Beverly, MA, USA, 1:1000), total mTOR (Cell signaling, 1:1000), which recognized at the same time mTORC1 and mTORC2, phosphorylated P70S6K at threonine 389 (Cell signaling, 1:1000), total P70S6K (Cell signaling, 1:1000), phosphorylated 4EBP1 at threonine 37 and 46 (Cell signaling, 1:1000), total 4EBP1 (Cell signaling, 1:1000) and GAPDH (Abcam, Cambridge, MA, USA 1:20000). Membranes were washed and incubated with corresponding anti-mouse or anti-rabbit secondary horseradish peroxidase antibodies (Cell signaling, 1:5000) diluted in 1% BSA/TBS-Tween 0.1% for 1 h at room temperature. To visualize protein bands, chemiluminescence reactions were used (SuperSignal West Femto chemiluminescence kit, Pierce Chemical Co, Rockford, IL, USA). Quantification was performed using the Visonwork LS software (UVP bioimaging Upland, CA, USA) and analyses of densitometry were expressed as relative optical density.

## STATISTICAL ANALYSIS

Data were analyzed with GraphPad Prism software (version 5.0, Graph Pad Software, San Diego, CA, USA). Statistical analysis was determined by unpaired Student’s *t*-test, one-way analysis of variance (ANOVA) followed by the Newman–Keuls *post hoc* test or two-way repeated-measures ANOVA followed by the Bonferroni *post hoc* test. Data are shown as the mean ± S.E.M and statistical significance was set at *P* < 0.05.

## RESULTS

### ROTAROD LEARNING INDUCED mTOR ACTIVATION

**FIGURE 2:**
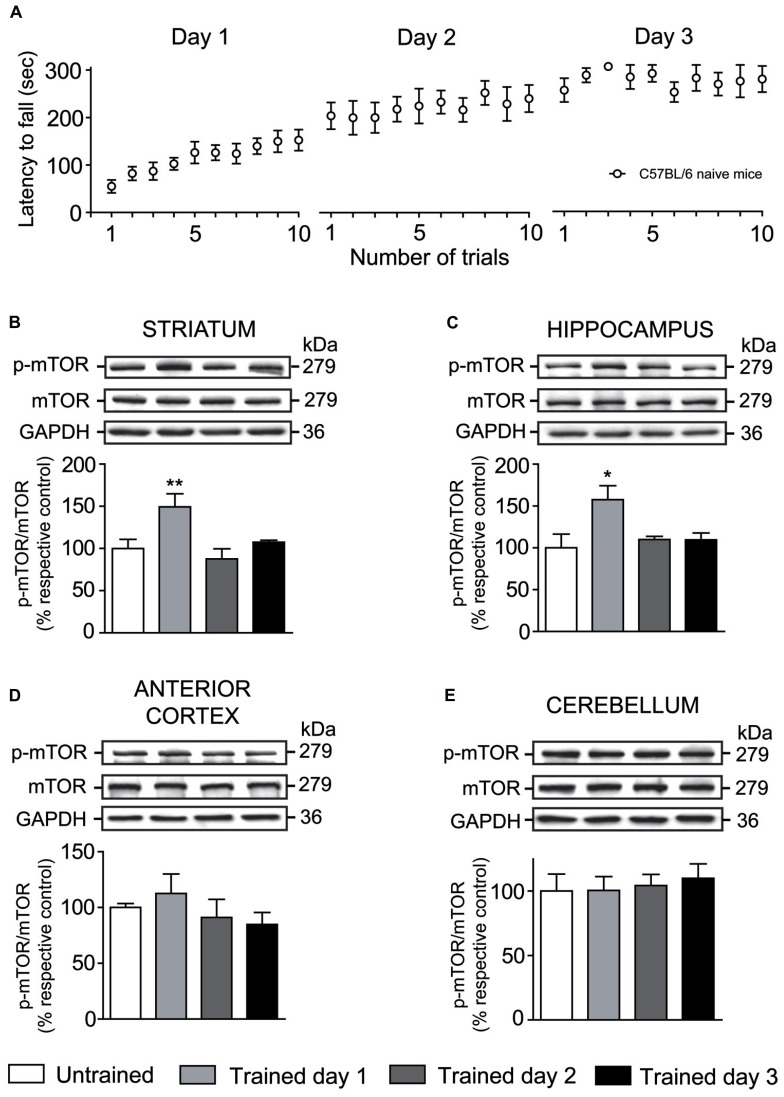
**Levels of phosphorylated mTOR is modulated by rotarod training in specific brain regions. (A)** Latency to fall on the accelerating rotarod for each trial at days 1, 2, and 3. Levels of total mTOR and phosphorylated mTOR were evaluated by western blot in the **(B)** striatum, **(C)** hippocampus, **(D)** anterior cortex, and **(E)** cerebellum of trained mice at days 1, 2, and 3. The data, expressed relative to levels of total mTOR, represent the mean of relative optical density of phosphorylated mTOR (expressed as a percentage of control values) ± S.E.M, *n* = 3 or 4; triplicate experiments for each mouse/group. **P < 0.01 and *P < 0.05 vs. respective untrained group.

As we previously published, the rotarod task illustrates the typical phases of motor skill learning (Bureau et al., 2010). At the first day of training, mice enhanced rapidly their performance over each trial corresponding to the early phase of learning. At the second day, mice still improved their scores but to a much lower extent, finally reaching a plateau on the third rotarod training day. The second and third days of rotarod training illustrates the consolidation phase of learning (**Figure 2A**).

In order to determine whether mTOR was activated during rotarod training, levels of total mTOR as well as levels of phosporylated mTOR at serine 2448 were assessed at days 1, 2, and 3 in the striatum, hippocampus, anterior cortex, and cerebellum. One-way ANOVA followed by the Newman–Keuls *post hoc* test was performed. At the first day of rotarod training, levels of phosphorylated mTOR were significantly increased in the striatum (*F*_(3,15)_ = 6.17, *P* < 0.01) and hippocampus (*F*_(3,15)_ = 4.29, *P* < 0.05), but were equal to those of untrained mice at the second and third days of training (**Figures 2B,C**). Levels of phosphorylated mTOR in the anterior cortex (*F*_(3,11)_ = 0.84, *P* = 0.51) and cerebellum (F_(3,15)_ = 0.17, *P* = 0.91) of trained mice were not significantly different to the untrained mice at any training day (**Figures 2D,E**). In all brain structures studied, levels of total mTOR were unaffected by rotarod training (**Figures 2B–E**).

### EFFECT OF SYSTEMIC AND INTRASTRIATAL INHIBITION OF mTOR ON ROTAROD LEARNING

**FIGURE 3:**
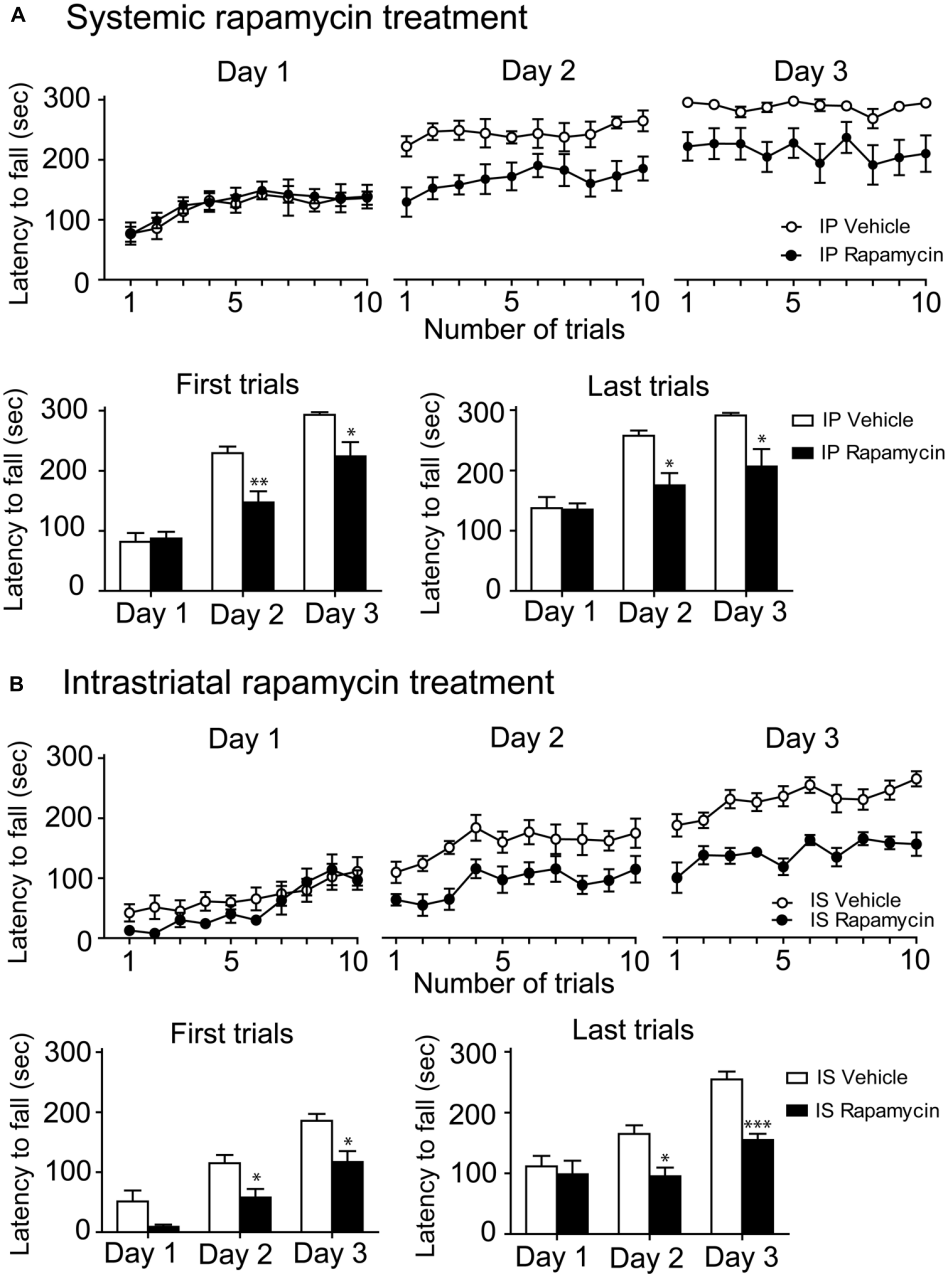
**Systemic and intrastriatal inhibitions of mTOR by rapamycin impaired motor learning. (A)** Time spent by vehicle (DMSO) and intraperitoneal (IP) rapamycin-treated mice on the accelerating rotarod for each trial at days 1, 2, and 3. Rotarod learning was analyzed by pooling together the two first or the two last trials/training day of both groups. Values represent the average mean latency to fall expressed in seconds ± S.E.M, *n* = 8 vehicle-treated mice and *n* = 12 rapamycin-treated mice. **P < 0.01 and *P < 0.05 vs. respective vehicle-treated group. **(B)** Time spent by vehicle (DMSO) and intrastriatal (IS) rapamycin-treated mice on the accelerating rotarod for each trial at days 1, 2, and 3. Rotarod learning was analyzed by pooling together the two first or the two last trials/training day of both groups. Values represent the average mean latency to fall expressed in seconds ± S.E.M, *n* = 7 vehicle-treated mice and *n* = 4 rapamycin-treated mice. ***P < 0.001 and *P < 0.05 vs. respective vehicle-treated group.

To investigate whether mTOR activity is involved in the learning of a complex motor task, the mTOR inhibitor rapamycin was systemically injected in mice before every single rotarod training. It is noteworthy that it was previously believed that a rapamycin treatment could discriminate mTORC1 and mTORC2 (mTORC2 was considered rapamycin-resistant), but recent studies recognized that rapamycin inhibited both complexes (Lamming et al., 2012). On the first training day, we observed a rapid improvement of performances in both rapamycin-treated and control mice. However, on the second and third day, the performances of rapamycin-treated mice were significantly lower than control mice (**Figure 3A**). Statistical analysis of the two first and two last trials of each training session were achieved using a two-way repeated measures ANOVA followed by the Bonferroni *post hoc* test. Analysis of the first trials displayed a significant difference in treatments (*F*_(1,34)_ = 7.54, *P* < 0.05), an effect of training days (*F*_(2,34)_ = 57.95, *P* < 0.001) and an interaction between treatments and training days (*F*_(2,34)_ = 4.23, *P* < 0.05; **Figure 3A**). Analysis of the last trials indicated a significant effect of treatment (*F*_(1,34)_ = 6.96, *P* < 0.05), training days (*F*_(2,34)_ = 21.63, *P* < 0.001) as well as an interaction between treatments and training days (*F*_(2,34)_ = 3.53, *P* < 0.05; **Figure 3A**).

We next investigated the effect of mTOR inhibition directly in the dorsal striatum of mice during the rotarod learning. We observed that performances of rapamycin-treated and control mice were similar to those obtained with the systemic approach (**Figure 3B**). Statistical analysis of the two first trials revealed a significant difference in treatments (*F*_(1,18)_ = 12.24, *P* < 0.01) and an effect of training days (*F*_(2,18)_ = 42.31, *P* < 0.001), but no interaction between the treatments and the training days (*F*_(2,18)_ = 0.50, *P* > 0.05; **Figure 3B**). Analysis of the two last trials indicated a significant effect of treatment (*F*_(1,18)_ = 21.07, *P* < 0.01) and training days (*F*_(2,18)_ = 19.27, *P* < 0.001), although no interaction between the treatments and the training days was observed (*F*_(2,18)_ = 3.45, *P* > 0.05; **Figure 3B**).

**FIGURE 4:**
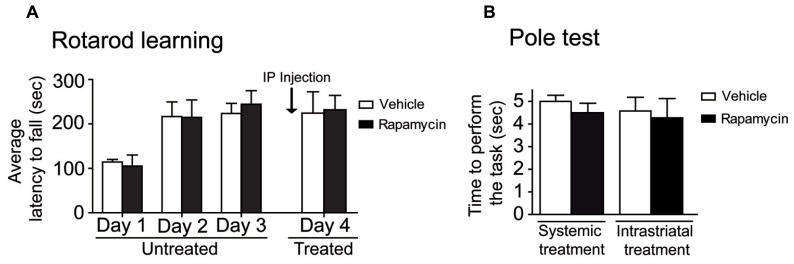
**Motor abilities are not affected in mice after mTOR inhibitions. (A)** Mice were trained on the accelerating rotarod during 4 days. At day 4, mice were systemically injected with either vehicle (DMSO) or rapamycin. Data represent the average mean latency to fall expressed in seconds from 10 assays per training day ± S.E.M, *n* = 3–4 mice/group. **(B)** Motor abilities in systemic and intrastriatal rapamycin-treated mice were assessed by the pole test and compared to the vehicle (DMSO) treated mice. Data represent the time required to perform the task in seconds ± S.E.M, *n* = 3–12 mice/groups.

To investigate the effect of systemic rapamycin injections on motor activity endurance, mice were trained on the rotarod during four days, including 10 assays per day. At the fourth day, mice were treated with a systemic injection of either vehicle or rapamycin. No difference was observed in rotarod performances between rapamycin and vehicle-treated mice. Statistical analysis, using a two-way repeated measures ANOVA followed by the Bonferroni *post hoc* test, revealed no significant difference in treatments (*F*_(1, 15)_ = 0.03, *P* > 0.05), a significant effect in training days (*F*_(3,15)_ = 23.57, *P* < 0.001) and no interaction between treatments and training days (*F*_(3, 15)_ = 0.22, *P* > 0.05; **Figure 4A**). To verify whether the systemic or intrastriatal rapamycin treatments interfered with motor abilities, the pole test was performed. A similar amount of time was required to perform the task in systemic (Unpaired *t*-test, *P* = 0.38) and instrastriatal (Unpaired *t*-test, *P* = 0.78) rapamycin-treated mice compared to their respective control (**Figure 4B**).

### RAPAMYCIN TREATMENTS REDUCED THE LEVELS OF PHOSPHORYLATED P70S6K AND 4EBP1 IN THE STRIATUM

**FIGURE 5:**
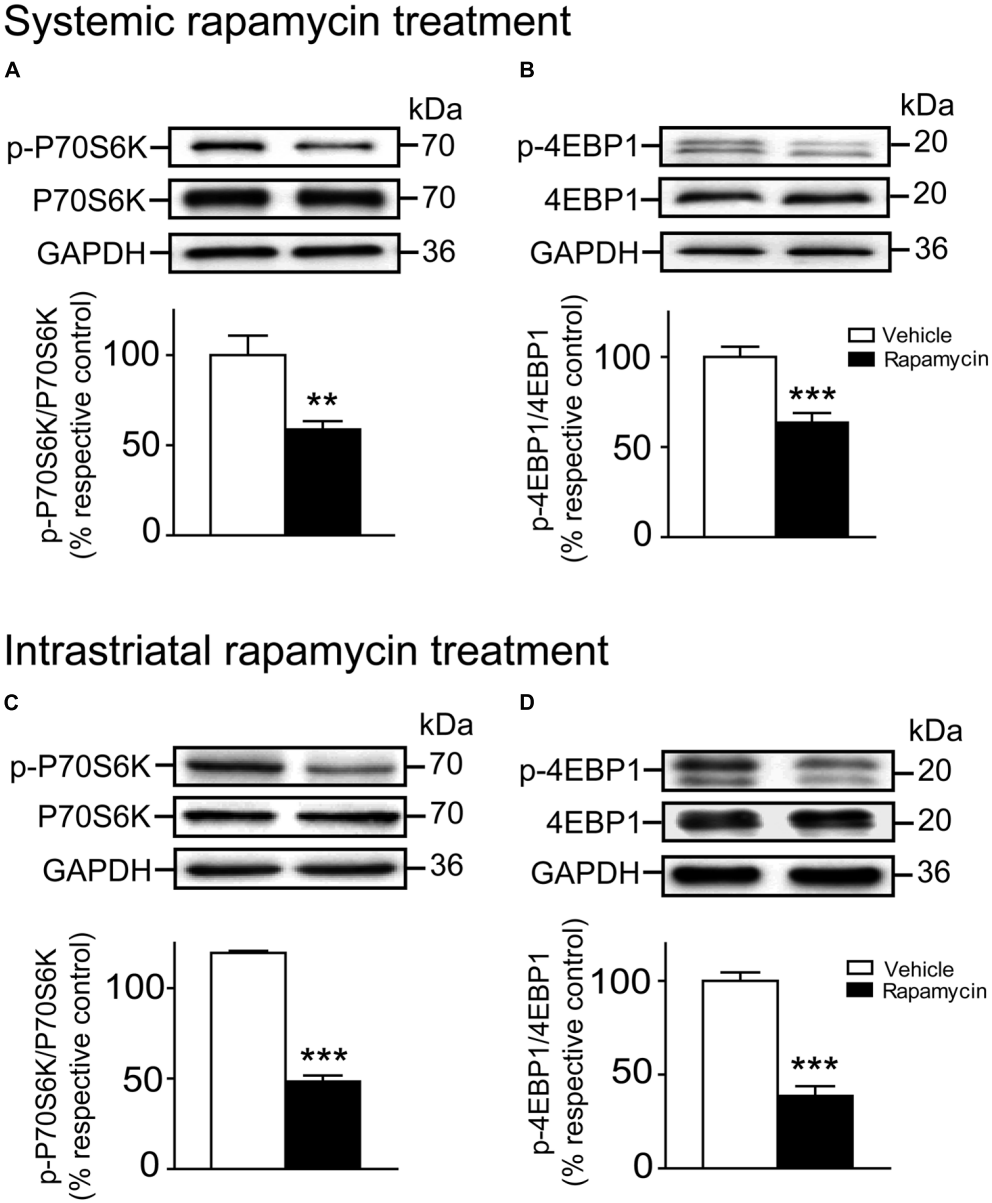
**Systemic and intrastriatal rapamycin treatments decreased the phosphorylation levels of striatal mTORC1 downstream substrates.** Levels of total and phosphorylated **(A)** P70S6K and **(B)** 4EBP1 were evaluated by western blot after systemic rapamycin treatments. The data, expressed to relative total P70S6K or 4EBP1 levels, represent the mean of relative optical density of phosphorylated P70S6K or 4EBP1 (expressed as a percentage of control values) ± S.E.M, *n* = 8–12 vehicle (DMSO) and rapamycin-treated mice; triplicate experiments for each mouse/group. ***P < 0.001 and **P < 0.01 vs. vehicle-treated group. Levels of total and phosphorylated **(C)** P70S6K and **(D)** 4EBP1 were also evaluated following instrastriatal rapamycin treatments. The data, expressed to relative total P70S6K or 4EBP1 levels, represent the mean of relative optical density of phosphorylated P70S6K or 4EBP1 (expressed as a percentage of control values) ± S.E.M, *n* = 3–4 vehicle (DMSO) and rapamycin-treated mice; triplicate experiments for each mouse/group. ***P < 0.001 vs. vehicle-treated group.

The levels of P70S6K and 4EBP1, two mTORC1 downstream substrates, as well as the levels of their phosphorylated form were evaluated following rapamycin treatments. We observed that the levels of phosphorylated P70S6K at Thr389 (Unpaired *t*-test, *P* < 0.01) and 4EBP1 at Thr37/46 (Unpaired *t*-test, *P* < 0.001) were strongly reduced after the intraperitoneal rapamycin treatments (**Figures 5A,B**). Likewise, levels of phosphorylated P70S6K (Unpaired *t*-test, *P* < 0.001) and 4EBP1 (Unpaired *t*-test, *P* < 0.001) were reduced after the intrastriatal rapamycin treatments (**Figures 5C,D**). Interestingly, both treatments did not affect the levels of total P70S6K and 4EBP1 (**Figures 5A–D**).

### INTRASTRIATAL INFUSION OF mTOR siRNA ALTERED ROTAROD LEARNING AND DECREASED THE PHOSPHORYLATION LEVELS OF STRIATAL mTORC1 DOWNSTREAM SUBSTRATES

**FIGURE 6:**
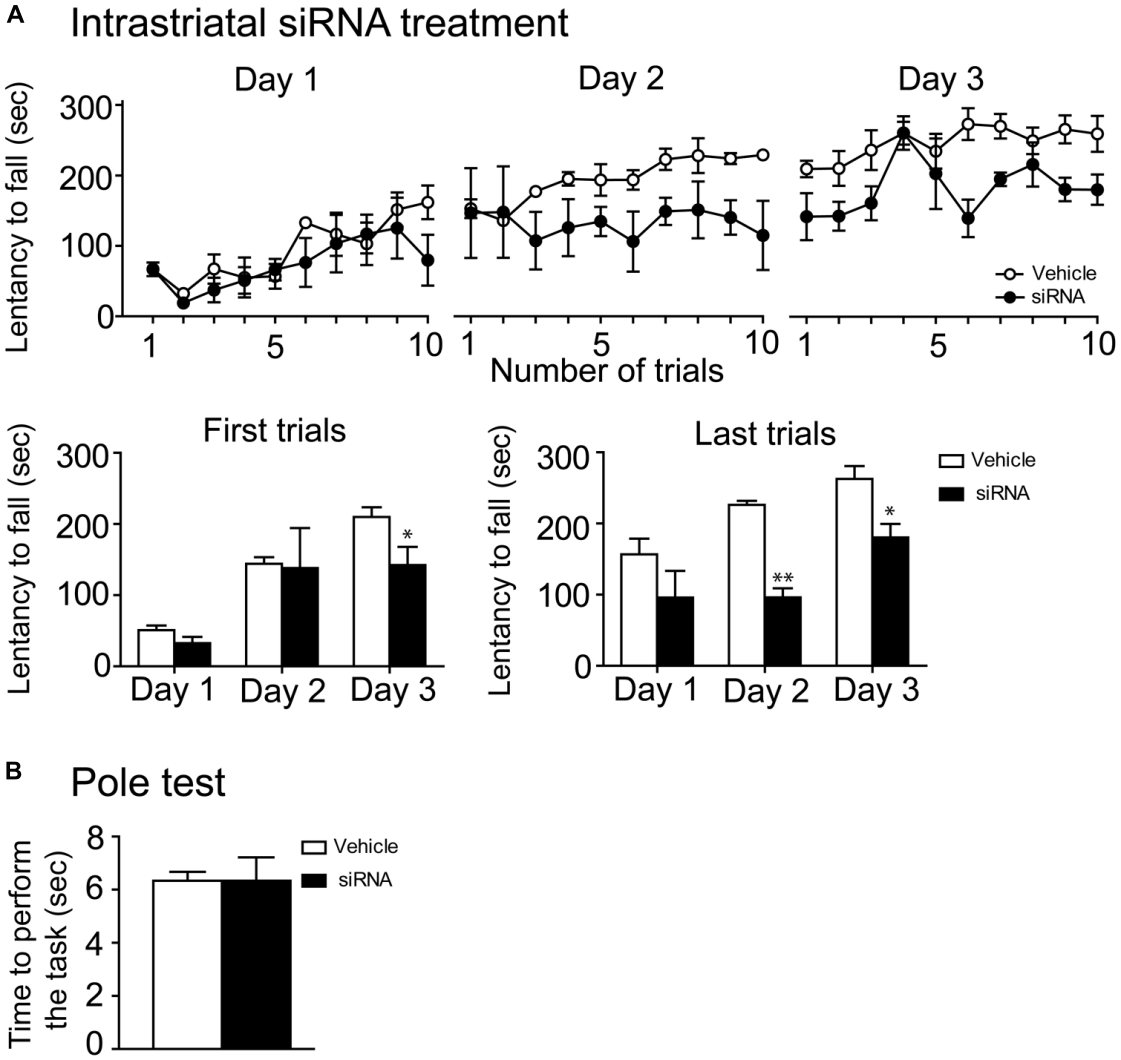
**mTOR siRNA infusion impaired rotarod learning. (A)** Time spent by vehicle (distillated/deionized water DNase/RNase free) and siRNA-treated mice on the accelerating rotarod for each trial at days 1, 2, and 3. Rotarod learning was analyzed by pooling together the two first or the two last trials/training day of both groups. Data represent the average mean latency to fall expressed in seconds ± S.E.M, *n* = 3–4 vehicle-treated and siRNA-treated mice. **P < 0.01 and *P < 0.05 vs. respective vehicle-treated group. **(B)** Motor abilities of mTOR siRNA-treated mice were also assessed by the pole test and compared to their respective controls. Data represent the time required to perform the task in second ± S.E.M, *n =* 3–4 mice/groups.

In order to further confirm the implication of mTOR in motor skill learning, we infused directly into the dorsal striatum a specific mTOR siRNA during 14 consecutive days. On the 12th, 13th, and 14th days, we assessed the latency to fall on the accelerating rotarod. During the first day of training, there were no difference in the performances of siRNA and vehicle-treated mice. However, performances of the siRNA-treated mice were lower on the second and third day, when compared to their respective controls (**Figure 6A**). Statistical analyses of the two first and the two last trials of every rotarod training day were performed using a two-way repeated measures ANOVA followed by the Bonferroni *post hoc* test. The analysis of the two first trials revealed no significant difference in treatments (*F*_(1,10)_ = 1.89, *P >* 0.05), a significant effect in training days (*F*_(2,10)_ = 23.08, *P* < 0.001) and no interaction between treatments and training days (*F*_(2,10)_ = 1.26, *P* > 0.05; **Figure 6A**). However, statistical analysis of the two last rotarod trials displayed a significant effect of siRNA treatment (*F*_(1,10)_ = 24.41, *P* < 0.01) and training days (*F*_(2,10)_ = 12.37, *P* < 0.01), although no interaction between siRNA treatment and training days was observed (*F*_(2,10)_ = 1.7, *P >* 0.05; **Figure 6A**). The siRNA-treated mice were also tested with the pole test and no significant difference between these mice and the vehicle-treated mice was observed (Unpaired *t*-test, *P* = 1.00; **Figure 6B**).

**FIGURE 7:**
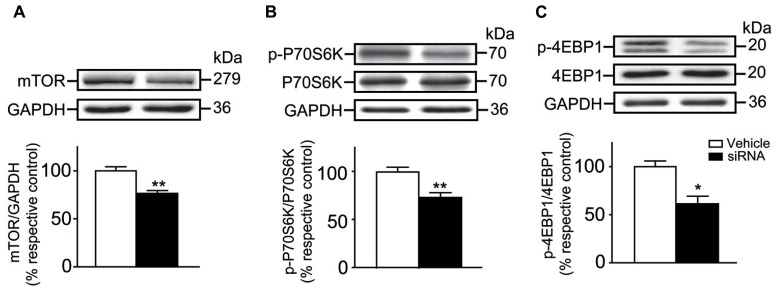
**mTOR siRNA infusion inhibited striatal mTOR expression and decreased the levels of phosphorylated P70S6K and 4EBP1. (A)** Levels of total striatal mTOR were evaluated by western blot after the infusion of vehicle (distillated/deionized water DNase/RNase free) or mTOR siRNA. The data, expressed relative to GAPDH, represent the mean of relative optical density of total mTOR (expressed as a percentage of control values) ± S.E.M, *n =* 3–4; triplicate experiments for each mouse/group. **P < 0.01 vs. vehicle-treated group. Levels of total and phosphorylated **(B)** P70S6K and **(C)** 4EBP1 were also evaluated. The data, expressed to relative levels of total P70S6K or 4EBP1, represent the mean of relative optical density of phosphorylated P70S6K or 4EBP1 (expressed as a percentage of control values) ± S.E.M, *n* = 3–4 vehicle-treated and siRNA-treated mice; triplicate experiments for each mouse/group. **P < 0.01 and *P < 0.05 vs. vehicle-treated group.

The effect of siRNA infusion on the levels of striatal mTOR, P70S6K and 4EBP1 were verified. We observed that levels of total mTOR were decreased by ~25% in the siRNA-treated mice when compared to vehicle-treated mice (Unpaired *t*-test, *P* < 0.01; **Figure 7A**). This result confirmed that our experiment design was sound considering that previous work using siRNA injections in the striatum of a living mouse revealed a reduction of the targeted proteins within the range of 40% (Thakker et al., 2004). Interestingly, levels of phosphorylated P70S6K (Unpaired *t*-test, *P* < 0.01) and 4EBP1 (Unpaired *t*-test, *P* < 0.05) were also significantly decreased compared to vehicle-treated mice (**Figures 7B,C**). No difference in levels of total P70S6K and 4EBP1 was observed in both groups of mice (**Figures 7A–C**).

## DISCUSSION

At the behavioral level, our study demonstrates that inhibition of mTOR activity impaired significantly motor learning. We believe these effects on motor behavior are reminiscent of a learning deficit as our findings argue against a role of mTOR in motor capacity. The first evidence is the fact that a reduced mTOR activity does not affect the performances of mice at the pole test. In addition, peripheral rapamycin injections in mice that have fully learned the accelerating rotarod task have no effect. This particular finding is supported by a previous study demonstrating that systemic administrations of rapamycin do not affect mice performances when they are pre-trained on the rotarod (Deli et al., 2012). Moreover, the fact that both systemic and intrastriatal treatments lead to similar results suggests that possible effects of mTOR inhibition on peripheral functions such as muscles strength are unlikely. It is noteworthy that motor skill learning is assumed to be the major component responsible for the improvement in performance observed during rotarod training. The distinctive characteristic of the rotarod task is its independence to reward, spatial and instrumental features (Buitrago et al., 2004; Luft and Buitrago, 2005). Falling off the rod may represent a weak aversive stimulus. However, the distance to fall is small (40 cm) and we avoid additional aversive stimuli such as electric shocks. Taken together, it becomes evident that mTOR activities do not alter motor capacity but rather govern motor skill learning.

Our data demonstrate a differential involvement of brain structures during the acquisition of the rotarod task. In the striatum and hippocampus, mTOR activity is enhanced during the first day of rotarod training whereas no effect is noticed in the anterior cortex or the cerebellum. This is somewhat intriguing because these two last structures are also known to be engaged during the learning of a complex motor skill (Doyon and Benali, 2005; Bureau et al., 2010). We, and other groups, have previously documented that the participation of specific molecular pathways in motor skilled learning are differentially regulated over time and by the numbers of task repetitions (Yin et al., 2009; Bureau et al., 2010; D’Amours et al., 2011; Lemay-Clermont et al., 2011; Shiflett and Balleine, 2011). For instance, levels of phosphorylated ERK 1/2 were selectively induced in parallel with an upregulation of c-Fos in the cerebellum, motor cortex, cingulate cortex and dorsal striatum during the consolidation phase (Bureau et al., 2010). It is conceivable that a transitory surge of mTOR activity occurs in the cortex and cerebellum, which was returned to normal levels at the time we sacrificed the animal. Our data in the hippocampus are interesting because previous studies have demonstrated that it is not considerably engaged during the rotarod training, despite its recognized role in long-term memory and spatial learning (Bureau et al., 2010). It would be interesting to investigate whether this increase of mTOR levels after the first session of rotarod is due to motor skill learning or to hippocampal dependent behaviors such as spatial learning or object recognition. Further analysis is ultimately needed to conclude on the role of mTOR in the anterior cortex, the cerebellum or the hippocampus. Nonetheless, using rapamycin and siRNA treatments, we demonstrate that mTOR of the dorsal striatum is mandatory to motor skill learning. Previous studies have demonstrated the importance of the striatum structure in rotarod training. For instance, rotarod training is completely abolished in the striatum specific NMDAR1 knockout mice (Dang et al., 2006). Interestingly, in our experiments, rapamycin and siRNA treatments do not completely prevent the improvement of performances during rotarod trainings. As no previous study has directly assessed the role of mTOR in motor behavior, it is difficult to compare our data with the literature. It has been shown that mice with genetic deletion of the mTOR downstream target 4EBP2 display low performances at every session of the accelerating rotarod trainings (Banko et al., 2007). However, impaired motor coordination and balance were suspected in this study, but no other tests of motor abilities were performed to discriminate motor execution from motor learning. Many causes could account for an incomplete effect of mTOR inhibitions. Rapamycin and siRNA treatments seem to provide only a partial inhibitory effect on striatal mTOR activity, as measured by the protein levels of the mTOR targets P70S6K and 4EBP1. Adaptive mechanisms may perhaps compensate and counterbalance the reduction in mTOR activity. This is a very interesting idea that merit to be investigated in future. At some point, we believe it will be possible to establish a heuristic model of the mTOR activation at different sites of the brain and understand how these pathways are elaborate in, or the result of, long-term motor learning.

The present study demonstrates that striatal mTOR activity has no influence in the early phase of learning but interferes with the later phases during the accelerating rotarod training, which are known to be associated with consolidation processes (Luft and Buitrago, 2005). Notably, mTOR inhibitions have been shown to alter memory consolidation and the long-term memory processes of other types of learning task such as non-reinforced retrieval of inhibitory avoidance conditioning (a fear-motivated memory task), in discrimination of complex auditory stimuli as well as in a fear conditioning task (Schicknick et al., 2008; Sui et al., 2008; Jobim et al., 2011a,b). A role for mTOR in general memory processes is predictable based on the numerous published studies on this topic (Jernigan et al., 2011; Talos et al., 2012; Chandran et al., 2013), but its implication in the encoding processes associated with long-term motor memory is novel. During the learning of a complex motor task, short-term improvements occur over repeated running trials within the first session (intrasession). Long-term improvements (intersession) develop over two to three sessions with rest periods between each session. This later type of memory stabilization involves neuronal processing after the training that transfers the memory from an unsteady state to a more stable state (Yin et al., 2009). Our data demonstrate that phosphorylation levels of mTOR are enhanced at the end of the first session, whereas they are not affected on the following sessions. This indicates that mTOR protein activation is necessary to initiate the molecular sequence leading to memory stabilization during rotarod training. Further experiments would be necessary to investigate how mTOR activation could influence molecular mechanisms of motor memory from an unsteady state to a more stable state. Our findings do propose, however, that the activity of two downstream molecular targets of mTORC1, P70S6K, and 4EBP1, are clearly involved. Such as demonstrated previously *in vitro* and *in vivo* (Sarbassov et al., 2005; Wang and Proud, 2006; Hoeffer and Klann, 2009; Urbanska et al., 2012), we observed a reduction in the levels of phosphorylated P70S6K and phosphorylated 4EBP1 following systemic or intrastriatal injections of rapamycin as well as intrastriatal genetic knockdown of mTOR. Therefore, depressing downstream molecular targets of mTORC1 is associated with impaired memory consolidation during the learning of a complex motor skill.

By using pharmacological and siRNA tools, in the dorsal striatum, we have demonstrated that mTOR activation play a significant role in motor learning processes. In particular, we established that mTORC1 activation via the phosphorylation of P70S6K and 4EBP1 is one important molecular pathway implicated in the consolidation of the learning, but not the execution capacity of the accelerating rotarod skill. Understanding the relationship between molecular processes and motor adaptive behavior is an important but daunting goal in this research field. We believe our study makes one step further toward a better understanding of proteins interaction, organization and function and this will lead to a global view of the role of synapses in the control and learning of motor behaviors.

## AUTHOR CONTRIBUTIONS

Yan Bergeron conducted this research. Yan Bergeron, Geneviève Bureau, and Laure Chagniel executed surgical procedures. Yan Bergeron and Michel Cyr wrote the manuscript. Michel Cyr supervised the project. Michel Cyr and Guy Massicotte contributed to the conceptual frame of the study and edited the manuscript.

## Conflict of Interest Statement

The authors declare that the research was conducted in the absence of any commercial or financial relationships that could be construed as a potential conflict of interest.
